# Possible involvement of normalized Pin1 expression level and AMPK activation in the molecular mechanisms underlying renal protective effects of SGLT2 inhibitors in mice

**DOI:** 10.1186/s13098-019-0454-6

**Published:** 2019-07-22

**Authors:** Masa-Ki Inoue, Yasuka Matsunaga, Yusuke Nakatsu, Takeshi Yamamotoya, Koji Ueda, Akifumi Kushiyama, Hideyuki Sakoda, Midori Fujishiro, Hiraku Ono, Misaki Iwashita, Tomomi Sano, Fusanori Nishimura, Kenichi Morii, Kensuke Sasaki, Takao Masaki, Tomoichiro Asano

**Affiliations:** 1 Department of Medical Science, Graduate School of Medicine, University of Hiroshima, 1-2-3 Kasumi, Minami-ku, Hiroshima City, Hiroshima 734-8551 Japan; 2 Division of Diabetes and Metabolism, The Institute for Adult Diseases, Asahi Life Foundation, Chuo-ku, Tokyo, 103-0002 Japan; 3 Center for Translational Research in Infection & Inflammation, School of Medicine, Tulane University, 6823 St. Charles Avenue, New Orleans, LA 70118 USA; 4 Division of Neurology, Respirology, Endocrinology and Metabolism, Department of Internal Medicine, Faculty of Medicine, University of Miyazaki, 5200 Kihara, Kiyotake, Miyazaki, 889-1692 Japan; 5 Division of Diabetes and Metabolic Diseases, Nihon University School of Medicine, Itabashi, Tokyo, 173-8610 Japan; 6 Department of Clinical Cell Biology, Graduate School of Medicine, Chiba University, 1-8-1 Inohana, Chuo-ku, Chiba City, Chiba 260-8670 Japan; 7 Section of Periodontology, Division of Oral Rehabilitation, Faculty of Dental Science, Kyushu University, Fukuoka, 3-1-1 Maidashi, Higashi-ku, Fukuoka City, Fukuoka 812-0054 Japan; 8 Department of Nephrology, Hiroshima University Hospital, 1-2-3 Kasumi, Minami-ku, Hiroshima City, Hiroshima 734-8551 Japan

**Keywords:** Diabetes mellitus, Nephropathy, SGLT2 inhibitor, Canagliflozin, AMPK, Pin1

## Abstract

**Background:**

Recently, clinical studies have shown the protective effects of sodium glucose co-transporter2 (SGLT2) inhibitors against progression of diabetic nephropathy, but the underlying molecular mechanisms remain unclear.

**Methods:**

Diabetic mice were prepared by injecting nicotinamide and streptozotocin, followed by high-sucrose diet feeding (NA/STZ/Suc mice). The SGLT2 inhibitor canagliflozin was administered as a 0.03% (w/w) mixture in the diet for 4 weeks. Then, various parameters and effects of canagliflozin on diabetic nephropathy were investigated.

**Results:**

Canagliflozin administration to NA/STZ/Suc mice normalized hyperglycemia as well as elevated renal mRNA of collagen 1a1, 1a2, CTGF, TNFα and MCP-1. Microscopic observation revealed reduced fibrotic deposition in the kidneys of canagliflozin-treated NA/STZ/Suc mice. Interestingly, the protein level of Pin1, reportedly involved in the inflammation and fibrosis affecting several tissues, was markedly increased in the NA/STZ/Suc mouse kidney, but this was normalized with canagliflozin treatment. The cells showing increased Pin1 expression in the kidney were mainly mesangial cells, along with podocytes, based on immunohistochemical analysis. Furthermore, it was revealed that canagliflozin induced AMP-activated kinase (AMPK) activation concentration-dependently in CRL1927 mesangial as well as THP-1 macrophage cell lines. AMPK activation was speculated to suppress mesangial cell proliferation and exert anti-inflammatory effects in hematopoietic cells.

**Conclusion:**

Therefore, we can reasonably suggest that normalized Pin1 expression and AMPK activation contribute to the molecular mechanisms underlying SGLT2 inhibitor-induced suppression of diabetic nephropathy, possibly at least in part by reducing inflammation and fibrotic change.

## Background

Recently, treatment objectives for diabetes mellitus have been advanced in accordance with the emergence of novel and potent hypoglycemic agents, which have made normalization of hyperglycemia easier than ever. Nevertheless, the most important and ultimate goals of diabetes treatment, the suppression of various diabetic complications and eventual extension of the life span with preserved quality of life, remain unchanged. Nephropathy, one of the three typical complications related to diabetes mellitus, is the most common cause of renal failure and can lead to the need for dialysis therapy [1]. Restriction of protein intake and administration of agents blocking the action of angiotensin II, in addition to the normalization of hyperglycemia, can markedly delay the progression of diabetic nephropathy [2, 3]. Further measures are still, however, necessary to reduce the incidence of progressing to diabetic nephropathy severe enough to require dialysis therapy.

Sodium glucose co-transporter2 (SGLT2) inhibitors are unique anti-diabetic drugs, since their mechanism of action involves excretion of excessive blood glucose into urine [4]. In the early period after their introduction, there was considerable concern regarding harmful effects on the kidney, since the estimated glomerular filtration rate (eGFR) is temporally reduced soon after the initiation of SGLT2 inhibitor administration [5]. However, to date, many clinical studies have shown SGLT2 inhibitors to block the progression of diabetic nephropathy in the long-term [6–10]. Treatment with canagliflozin was shown to be associated with decreased albuminuria and long-term preservation of eGFR [11]. Similar renal protective effects were reported in a clinical study using another SGLT2 inhibitor, dapagliflozin [12]. In addition, the potential molecular mechanisms for SGLT2 inhibition-mediated reno-protection were shown based on the in vitro findings using human proximal tubular cell lines treated with empagliflozin and canagliflozin [13]. Such renal protective effects of SGLT2 inhibitors were also observed in diabetic rodent models [14]. However, the molecular mechanisms underlying the favorable effects of SGLT2 inhibitors on the kidney have not been fully elucidated, though hypotheses have been put forward. One hypothesis is that an increased sodium concentration in tubular fluid causes opposite changes in single-nephron GFR via a tubuloglomerular feedback response. Another is that prevention of fibrosis and impaired proximal tubular functioning, by reducing glucose re-absorption and its accompanying ATP consumption, mediated by SGLT2 inhibitors contributes to the preservation of glomerular function. The effects exerted by blocking glucose re-absorption on the renin–angiotensin system and erythropoietin production also are possible mechanisms. Taken together, these observations suggest that multiple independent mechanisms may contribute to SGLT2 inhibitor-induced renal protective effects.

Prolyl isomerase Pin1 associates with the motif containing pSer-Pro or pThr-Pro, and isomerizes the proline residue in target proteins, thereby regulating their functions. To date, we and other research groups have revealed Pin1 to be highly involved in the development of metabolic syndromes, such as obesity, fatty liver and hypertension [15, 16]. Interestingly, Pin1 expressions are also known to be markedly upregulated, depending on nutrient conditions. However, the roles of Pin1 in the kidney have yet to be clarified.

Herein, we present the novel proposal that Pin1 and AMPK are involved in the protective action of SGLT2 inhibitors against diabetic nephropathy development.

## Methods

### Animals, diets and canagliflozin treatment protocols

Eight-week old C57BL/6J mice were purchased from CLEA Japan. After acclimation, both nicotinamide (120 mg/kg) and streptozotocin (100 mg/kg) were injected intraperitoneally into mice to induce relatively mild damage to pancreatic β cells. After 1 week, treated mice were randomly divided into two groups and were fed a high sucrose diet (HSD) with or without the SGLT2 inhibitor canagliflozin mixed, at 0.03% (w/w), into the diet. Control mice were fed a normal diet (ND) and all groups had free access to water and food.

All animals were handled in accordance with the guidelines for the care and use of experimental animals published by Hiroshima University.

### Reagent

Canagliflozin was provided by Mitsubishi Tanabe Pharma Corporation (Osaka, Japan). Streptozotocin and AICAR were purchased from Wako (Osaka, japan). Nicotinamide was purchased from SIGMA (St. Lewis, MO, USA). 2-Deoxyglucose (2-DG) was purchased from Tokyo Chemical Industries (Tokyo, Japan). Compound C was purchased from Calbiochem (CAS 866405-64-3). The cell culture reagents RPMI and DMEM were purchased from Nissui (Tokyo, Japan). Fetal bovine serum (FBS) was obtained from Biosera (Kansas City, MO, USA).

Antibodies were obtained from Cell Signaling Technology (pACC-11818S, ACC-3662S, pAMPK-4188S, AMPK-5832S, Cyclin D1-2922S, p-p70-9234S, p-70-2708S), R&D (PDGFRβ) and Santa Cruz (actin sc-47778 F1417, Pin-1sc46660 B0917).

### Immunohistochemistry

Paraffin-embedded kidney sections were subjected to Azan staining to detect fibrotic changes in renal glomeruli. For immunostaining of Pin1 or PDGFRβ, deparaffinized sections were treated as follows. Briefly, slides were incubated with 0.1% Triton solution for 5 min and then heated in 10 mM citrate (pH = 6.0) to activate the antigens. After being washed, the sections were incubated with anti-Pin1 or anti-PDGFRβ antibody at 4 °C overnight. After being washed with phosphate buffered saline (PBS), the slides were stained with a commercial diaminobenzidine staining kit.

### Cell culture

CRL1927 cells were maintained in Dulbecco’s modified Eagle’s medium (DMEM) containing 10% FBS at 37 °C in 5% CO_2_ in air. THP-1 cells were maintained in RPMI 1640 containing 10% FBS at 37 °C in 5% CO_2_ in air. For Pin1 knockdown, CRL1927 cells were treated with Negative siRNA or Pin1 siRNA, using RNAiMAX (Thermo Fisher Scientific, Tokyo, Japan). The sequences of siRNA are as follows.

Pin1-1: AGUAUUUAUUGUUCCUAAATT Pin1-2: CAGUAUUUAUUGUUCCUAATT.

### MTT assay

CRL1927 cells were cultivated at a concentration of 3 × 10^4^ cells/ml on collagen-coated 24-well plates. After being left overnight, canagliflozin was added to the culture medium at the indicated concentrations for 24 h. The cells were then incubated with DMEM containing 10% 3-(4,5-dimethylthiazol-2-yl)-2,5-diphenytletetrazolium bromide (MTT) for 4 h and absorbance was measured at 540 nm.

### ADP/ATP ratio assay

The ADP/ATP ratio was determined using an EnzyLight™ ADP/ATP ratio assay kit (BioAssay Systems, Hayward, CA, USA). CRL1927 cells were cultivated at a concentration of 3 × 10^4^ cells/ml in collagen-coated 24-well plates. After being left overnight, the samples were treated with canagliflozin (0, 2, 5 and 10 μM) for 1 h. The ADP/ATP ratio assay was then performed and luminosity was measured.

### Propidium iodide assay

Cultivated CRL1927 cells were treated with 100 μM canagliflozin or 100 μM H_2_O_2._ After 24 h, 10 μg/ml propidium iodide was added to the cells, which were then incubated for 1 h allowing the penetration of PI into the dead cells. After being washed with PBS, the cells were observed under a fluorescence microscope.

### Immunoblotting

Kidneys were homogenized in lysis buffer containing 50 mM Tris–HCl (pH = 7.4), 150 mM NaCl, 1 mM ethylenediaminetetraacetic acid, 1% Triton X-100, 1 mM NaF, 1 mM Na_3_VO_4_, and 1 mM phenylmethylsulfonyl fluoride. The lysates were incubated on ice for 30 min and then centrifuged at 15,000 rpm for 30 min at 4 °C. After adjusting the protein concentrations, the supernatants were mixed and boiled with sample buffer. Proteins from cell lines were directly solubilized with sample buffer (0.4M Tris-HCl, 8% SDS, 20% glycerol, 10% ME, 0.2% BPB). Proteins were separated by SDS-PAGE and then transferred to PVDF membranes. After being blocked with 3% bovine serum albumin, membranes were incubated with primary antibody (1:2000) for 1 h at room temperature (RT). Next, the membranes were washed with PBS three times and then reacted with secondary antibodies (1:4000) for 1 h at RT. After being washed, proteins were detected, using Supersignal West Pico Substrate (Thermo Scientific, Waltham, MA, USA) or ImmunoStar LD (Wako).

### Real-time PCR

Total RNA from cells or tissues was isolated using Sepasol reagent (Nacalai Tesque, Kyoto, Japan). First-strand cDNA was obtained using a Verso cDNA Synthesis Kit (Thermo Scientific), according to the kit instructions. This kit contains a reagent designed to exclude genomic DNA contamination. Real-time PCR was performed using the CFX96 real-time PCR system (Bio-Rad, Hercules, CA, USA) with KAPA SYBR Green. For experiments employing THP-1, the cells were pre-treated with canagliflozin for 30 min and then stimulated with 10 ng/ml of lipopolysaccharide (LPS) for 6 h. The primers used are shown in Table 1.

**Table 1:** The list of primer sequences

Gene	Forward primer	Reverse primer
mCTGF	CAAAGCAGCTGCAAATACCA	GGCCAAATGTGTCTTCCAGT
mCol1a1	GAGCGGAGAGTACTGGATCG	GCTTCTTTTCCTTGGGGTTC
mCol1a2	CCGTGCTTCTCAGAACATCA	GAGCAGCCATCGACTAGGAC
mIL-1b	CGTGGACCTTCCAGGATGAG	GCTCATATGGGTCCGACAGC
mIL-6	CCATCCAGTTGCCTTCTTGG	TGCAAGTGCATCATCGTTGT
mMCP1	AGGTCCCTGTCATGCTTCTG	TCTGGACCCATTCCTTCTTG
mTNFa	GAACTGGCAGAAGAGGCACT	AGGGTCTGGGCCATAGAACT
mTGFb1	TTGCTTCAGCTCCACAGAGA	TGGTTGTAGAGGGCAAGGAC
mF4/80	TCTGGGGAGCTTACGATGGA	TAGGAATCCCGCAATGATGG
mPin1	CGGCAGGAAAAGATCACCAG	TCCCCTGTCCGTAGAGCAAA
mGAPDH	TGATGGGTGTGAACCACG	GGGCCATCCACAGTCTTCTG

### Statistical analysis

The results were analyzed using EZR (Saitama Medical Center, Jichi Medical University, Saitama, Japan) [17]. Values are presented as mean ± SEM. Statistical significance was calculated employing Student’s unpaired *t*-test when comparing two groups, and one-way ANOVA followed by the post hoc Tukey’s test for multiple comparisons. In this study, we considered *P* < 0.05 to indicate a statistically significant difference.

## Results

### Treatment with canagliflozin suppressed diabetic nephropathy development and reduced the expressions of genes related to fibrosis and inflammation

**Fig. 1:**
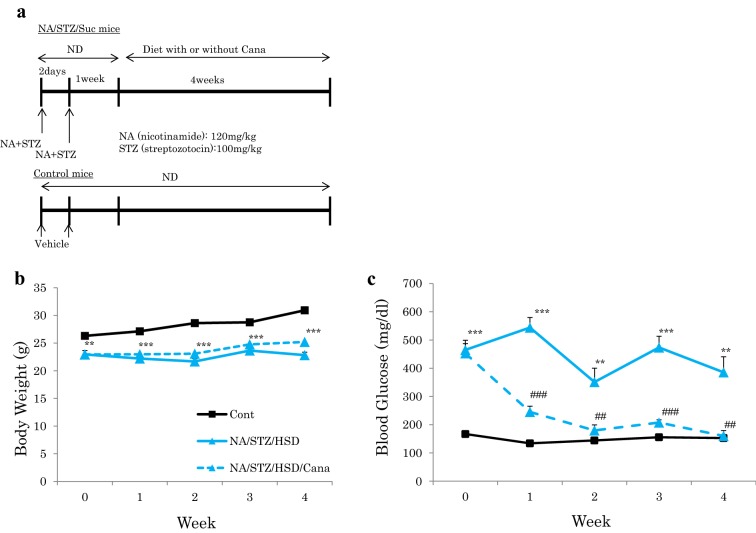
Administration of canagliflozin normalized hyperglycemia in NA/STZ/Suc mice. **a** Scheme of this study. **b**, **c** Body weights and blood glucose were measured once a week. (n = 8) *P < 0.05, **P < 0.01, ***P < 0.001

We created type 2 diabetic model mice by injecting both streptozotocin (120 mg/kg) and nicotinamide (100 mg/kg), to cause mild pancreatic β cell dysfunction, followed by HSD feeding to induce insulin resistance. These diabetic mice (NA/STZ/Suc mice) were randomly divided into two groups and treated with or without canagliflozin, respectively (Fig. 1a). Hyperglycemia was normalized in the former group. The body weights of NA/STZ/Suc mice were significantly lower than those of the controls because of diabetes mellitus. Treatment with canagliflozin did not affect body weight (Fig. 1b), but markedly normalized hyperglycemia in the NA/STZ/Suc mice (Fig. 1c).

**Fig. 2:**
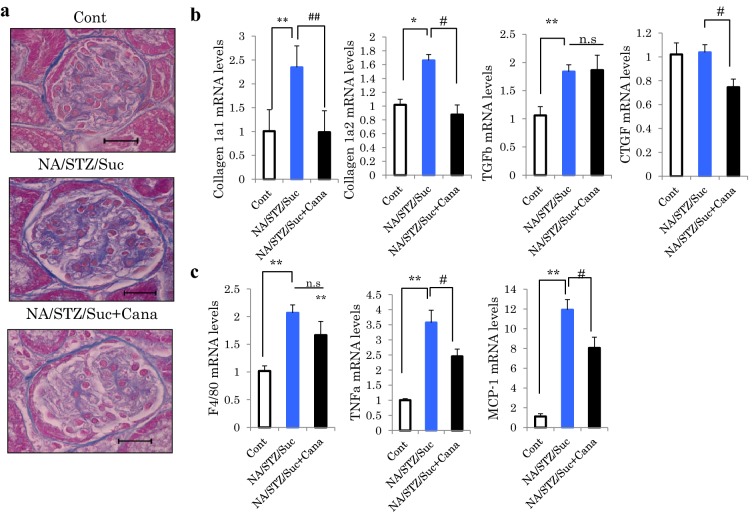
The expressions of fibrosis and inflammation markers were elevated in the NA/STZ/Suc mice, and were normalized by canagliflozin treatment. **a** AZAN staining of kidney sections. Scale bars: 50 μm. **b** Relative mRNA levels of fibrotic markers in the kidneys were investigated. (n = 5). **c** Relative mRNA levels of inflammatory cytokines in the kidneys. (n = 5) *^,#^P < 0.05, **^,##^P < 0.01, ***P < 0.001

Four weeks after the initiation of canagliflozin administration, the mice were sacrificed and the microscopic findings of the kidney with AZAN staining were compared among the groups. Microscopic observation revealed glomerular fibrosis in the kidney to be increased in the NA/STZ/Suc mice, as compared with the controls. Canagliflozin-treated NA/STZ/Suc mice generally had less collagen deposition than untreated NA/STZ/Suc mice (Fig. 2a).

To biochemically confirm the microscopic changes in collagen depositions observed in the kidneys of NA/STZ/Suc diabetic mice and their attenuation in the SGLT2 inhibitor treated mice, mRNA levels of collagen 1a1 and 1a2 were measured. NA/STZ/Suc mice showed markedly up-regulated collagen 1a1 and 1a2, both of which were normalized by canagliflozin treatment (Fig. 2b). In addition, although administration of canagliflozin had no impact on TGFβ expression levels, CTGF expression was reduced. Similarly, mRNA levels of F4/80, a marker of infiltrating macrophages, and those of inflammatory cytokines such as TNFα and MCP-1, were also elevated in NA/STZ/Suc mice. These elevations were partially normalized by canagliflozin treatment. On the whole, these results suggest canagliflozin to exert suppressive effects against inflammation and fibrosis in the kidney (Fig. 2c).

### Pin1 protein expression was elevated in the kidneys of diabetic model mice and was normalized by canagliflozin treatment

**Fig. 3:**
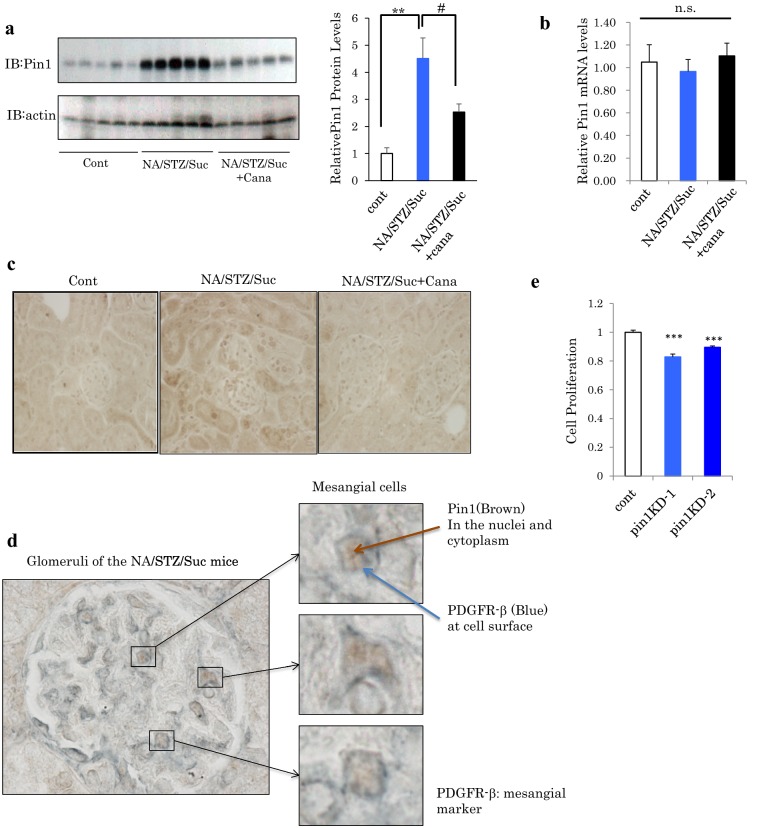
Renal Pin1 expression was markedly increased in the NA/STZ/Suc mice and was normalized by canagliflozin treatment. **a** Whole kidneys were extirpated from each mouse and Pin1 expression levels were examined by immunoblotting. (n = 5). **b** Relative mRNA levels of Pin1 in mouse kidneys. (n = 6). **c** Immunostaining of the renal sections with anti-Pin1 antibody. **d** Double immunostaining with anti-Pin1 (brown staining) and anti-PDGFRβ (blue staining as a mesangial marker). Scale bars: 50 μm. **e** Cell proliferation assessed by MTT assay in CRL1927 Pin1 KD cells. (n = 6). *^,#^P < 0.05, **P < 0.01, ***P < 0.001

Previous reports have indicated the involvement of Pin1 in the formation of fibrosis in diverse tissue types [18–20]. In addition, we previously reported Pin1 expression to be regulated by nutrient conditions [15]. Therefore, we investigated the Pin1 expression levels in the kidneys of normal and NA/STZ/Suc mice with and without canagliflozin treatment. Importantly, it was clearly demonstrated that renal Pin1 protein levels were markedly increased in the NA/STZ/Suc mice as compared with the normal mice (Fig. 3a). On the other hand, there was no change in the Pin1 mRNA expression level (Fig. 3b). Canagliflozin-treated NA/STZ/Suc mice showed reduced Pin1 protein levels, which as were closer to those of normal mice, as compared with non-treated NA/STZ/Suc mice. The Pin1 expression level in the normal mouse kidney is reportedly higher in the tubule than in the glomerulus, observations confirmed in this study (left panel of Fig. 3c) [19]. However, immunochemical analysis using anti-Pin1 antibody revealed Pin1 expression in the NA/STZ/Suc mouse kidneys to be elevated mainly in the glomerulus, though also slightly in the tubules (Fig. 3c). Magnified images of double staining with anti-Pin1 and anti-PDGFR-β, specific mesangial cell markers, revealed the cells with increased Pin1 expression in the diabetic condition included mesangial cells (Fig. 3d). Moreover, treatment of CRL1927 cells with Pin1 siRNA slightly, but significantly, suppressed cell proliferation (Fig. 3e).

### Canagliflozin activated AMPK in mesangial cells

It was recently reported that canagliflozin activates AMPK, exerting a direct effect in hepatocytes and vessel cells [21, 22]. In addition, increased Pin1 expression reportedly leads to suppressed AMPK activation via the association of Pin1 with the γ subunit of AMPK [23]. Since AMPK impacts numerous cellular functions including suppression of cell proliferation and inflammatory cytokine expressions, we examined the effects of canagliflozin on AMPK phosphorylation in the mesangial CRL1927 and the macrophage THP-1 cell lines.

**Fig. 4:**
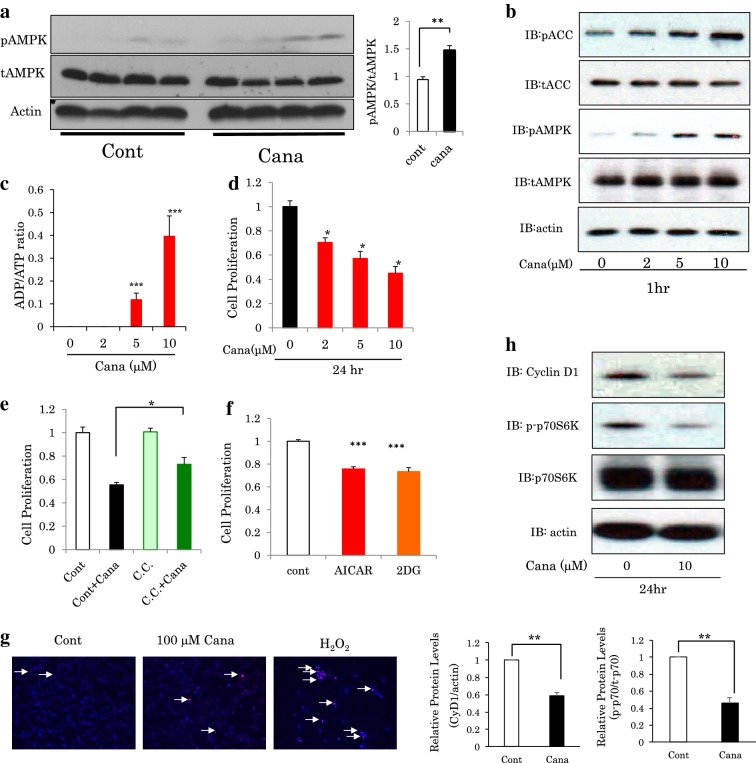
Canagliflozin activates AMPK and inhibits the proliferation of mesangial CRL1927 cells. **a** C57BL/6J mice were administered canagliflozin by intravenous injection. Two hours later, the kidneys were extirpated and lysates were immunoblotted using actin, anti-phosphorylated AMPK and anti-AMPK antibodies. (n = 4). **b** CRL1927 cells were treated without or with 2 μM, 5 μM and 10 μM canagliflozin for 1 h. The cell lysates were immunoblotted using anti-acetyl CoA carboxylase (pACC), anti-ACC, anti-phosphorylated AMPK and anti-AMPK antibodies. Representative data from three independent experiments are shown. **c** CRL1927 cells were exposed to canagliflozin at the indicated concentration for 1 h. Then, an ADP/ATP ratio assay was performed. (n = 5). **d** Cell proliferation assessed by MTT assay in CRL1927 cells cultured with 0 μM, 2 μM, 5 μM and 10 μM canagliflozin for 24 h. (n = 6). **e** Cell proliferation assessed by MTT assay in CRL1927 cells cultured with or without 5 μM compound C and 10 μM canagliflozin for 24 h. (n = 6). **f** Cell proliferation assessed by MTT assay in CRL1927 cells cultured with 5 mM 2DG and 0.5 mM AICAR for 24 h. (n = 6). **g** CRL1927 cells were exposed to 100 μM canagliflozin or hydroxyperoxide for 24 h. The cells were stained with 10 μg/ml propidium iodide for 1 h and then observed. **h** Canagliflozin was applied to CRL1927 cells for 24 h. Then, protein levels were examined. Representative data from two-independent experiments are shown. *P < 0.05, **P < 0.01

In the in vivo experiments, only a slight increase in AMPK phosphorylation in whole cell lysates of mouse kidney after venous injection of canagliflozin into tails (200 mg/kg) was detected, possibly because the kidney contains a wide range of cells including many not responsive to canagliflozin (Fig. 4a). In the in vitro experiments, AMPK and ACC were concentration-dependently phosphorylated in CRL1927 cells in response to canagliflozin, with no alteration in the protein amount of AMPK (Fig. 4b) [24]. The ADP/ATP ratio in mesangial cells was increased, canagliflozin-concentration dependently (Fig. 4c). The CRL1927 cell proliferation at the time point of 24 h after the addition of canagliflozin was also significantly suppressed (Fig. 4d). Addition of an AMPK activator, either 2-DG or AICAR, also decreased cell growth. Moreover, anti-proliferative effects exerted by canagliflozin were partially blocked by the AMPK inhibitor, compound C, suggesting the anti-proliferative effects of canagliflozin to be mediated, at least partially, by AMPK activation.

In contrast, numbers of apoptotic CRL1927 cells were unchanged with canagliflozin treatment, while being increased with H_2_O_2_ treatment (Fig. 4e). In addition, the levels of expression of the Cyclin D1 and phospho p70S6K proteins, involved in regulating cell proliferation and chemokine expression, were significantly decreased in canagliflozin-treated CRL1927 mesangial cells (Fig. 4f).

### Canagliflozin activated AMPK and suppressed cytokine expressions in THP-1 macrophages

**Fig. 5:**
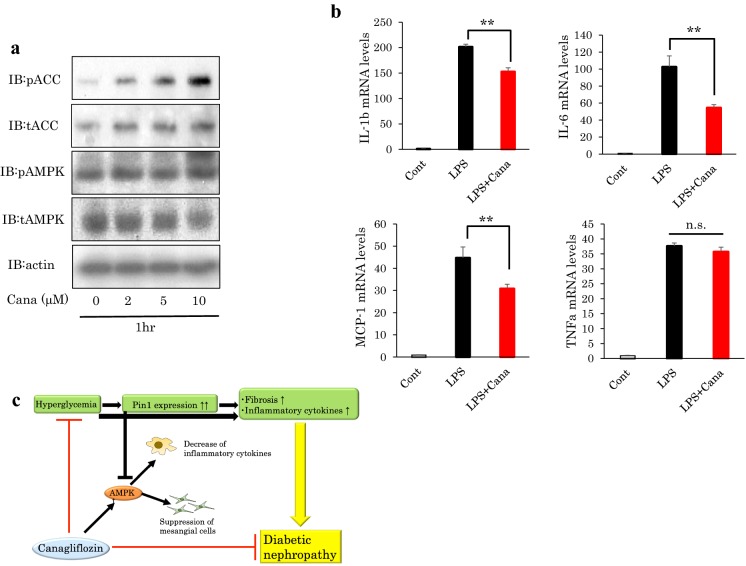
Canagliflozin also activates AMPK in macrophages. **a** THP-1 cells were treated with 0, 2, 5 and 10 μM canagliflozin for 1 h. Protein levels were examined with the indicated antibodies. Representative data from three-independent experiments are shown. **b** THP-1 cells were stimulated with 10 ng/ml LPS with or without 30 μM canagliflozin for 6 h. Then, RNA was extracted and expression levels of inflammatory cytokines were measured. (n = 5) **P < 0.01. **c** Proposed mechanisms of diabetic nephropathy development and canagliflozin-induced renal protective effect

Canagliflozin-induced AMPK and ACC phosphorylations were also clearly observed in THP-1 macrophages at canagliflozin concentrations of no less than 2 μM and were concentration-dependent (Fig. 5a). Canagliflozin suppressed the LPS-induced increases in mRNA expression levels of IL-1β, IL-6, and MCP-1, but did not significantly alter TNFα, in THP-1 cells (Fig. 5b).

## Discussion

Recent reports have revealed that SGLT2 inhibitors exert renal protective effects in diabetic rodent models [14]. Consistent with previous reports, our results also showed canagliflozin to improve the features of diabetic nephropathy. However, details of the underlying mechanisms remain controversial and several hypotheses have been put forward. In this study, we obtained data suggesting contributions of Pin1 and AMPK to the renal protective effects of SGLT2 inhibitors. Herein, we newly revealed that Pin1 protein in the mesangial cells of the kidney is also upregulated in the hyperglycemic state of NA/STZ/Suc mice, although there appeared some other unidentified cells with increased Pin1 expression. However, the expression levels of Pin1 mRNA did not differ among the three groups. We speculate that the changes in Pin1 protein expression levels in the kidneys are regulated by protein degradations.

Pin1 has been recognized as being involved in the pathogenesis of several diseases, including cancers and Alzheimer’s disease [25, 26]. In addition, previous reports described Pin1 as playing a critical role in the fibrotic processes of bleomycin-induced interstitial pneumonia [27], non-alcoholic hepatosteatosis [26] and phosphate-induced nephritis [20], and that all of these actions were blocked in Pin1 deficient mice. In terms of the kidney, macrophage infiltration and extracellular matrix accumulation in the interstitium, after feeding of a high phosphate diet, were reportedly suppressed in Pin1 null mice [20]. Unfortunately, to our knowledge, there is no murine model expressing CRE specifically in mesangial cells, and thus generation of mesangial cell-specific Pin1 KO mice was not feasible. Nevertheless, the report showing suppressed renal fibrosis in Pin1 KO mice fed a high-phosphate diet suggests that increased Pin1 in diabetic mice might also be involved in diabetic kidney impairment, making at least a limited contribution, and also that normalization of the Pin1 expression level by the treatment with an SGLT2 inhibitor is involved in its protective effect.

In the rat kidney, SGLT2 was reported to be expressed in proximal tubular epithelial cells and mesangial cells [28]. Canagliflozin inhibited high-glucose-induced activation of the protein kinase C (PKC)-NAD(P)H oxidase pathway and increased reactive oxygen species production in mesangial cells [29]. Canagliflozin also normalized the expression of TGF-β1, a key cytokine that mediates extracellular matrix accumulation and glomerular expansion in diabetes, and the expression of fibronectin, a predominant matrix protein in glomerular expansion in the mesangial cells of diabetes models [29]. Although canagliflozin did not decrease the mRNA expression level of TGF-β1 in the whole kidney in our study, the possibility that TGF-β1 expression was suppressed only in the mesangial cells expressing SGLT2 cannot be ruled out.

Pin1 PPIase activity was reportedly enhanced in human pulmonary eosinophils treated with a PKC-α agonist [30]. On the other hand, Pin1 inhibits PKC-α degradation by the proteasome. Therefore, PKC-α might be located upstream from Pin1 activation, while Pin1 raises cytosolic levels of PKC-α. In addition, Pin1 reportedly promotes the stability of TGF-β1 mRNA, which would drive fibroblast proliferation and extracellular matrix deposition. Taken together, these observations allow us to speculate that increased Pin1 in mesangial cells is involved in fibrosis via PKC and TGF-β1.

Another interesting finding is that canagliflozin induces AMPK activation in mouse kidneys, mesangial CRL1927 cells and THP-1 macrophages. This effect is likely to be physiological, since it is observed at canagliflozin concentrations of at least 2 μM and is concentration-dependent. Canagliflozin-induced AMPK activation was previously reported in the liver and vessel cells [21, 22]. It is possible that the canagliflozin-induced AMPK activation takes place only in a limited number of cell types such as mesangial cells, since renal cells are highly diverse. Pin1 strongly suppresses AMPK activation via its association with the γ subunit of AMPK without altering ATP levels [23]. On the other hand, the AMPK activation data obtained in this study by administering canagliflozin represent an acute in vitro response, mediated by direct inhibition of mitochondrial complex I by canagliflozin. AMPK activation reportedly induced numerous actions leading to the production of ATP and to reduced energy consumption. AMPK reportedly inactivates the mTOR pathway, thereby suppressing its downstream p70S6K and cyclin D1 [31]. As a result, cell cycle or protein production is attenuated by AMPK activation, in good agreement with our present data. Thus, it is possible that canagliflozin-induced AMPK activation contributes to the suppression of mesangial cell proliferation. In addition, activation of AMPK reportedly suppresses NF-κB and thereby reduces inflammatory cytokine expressions [32], which would eventually contribute to the tissue protective effects. Furthermore, increased Pin1 expression reportedly suppresses AMPK activation via the association of Pin1 with the γ subunit of AMPK. In the mesangial cells of diabetic mice, increased Pin1 might inhibit AMPK and decreased Pin1 by canagliflozin might activated AMPK, accounting for the contribution of canagliflozin to the amelioration of diabetic nephropathy. This is the first report, to our knowledge, to raise the possibility of Pin1 and AMPK involvement in the SGLT2 inhibitor-mediated protection against the development of diabetic kidney disease.

We cannot separate the reno-protective effects exerted by canagliflozin from those due to improved glycemic control or SGLT2-inhibition. Nevertheless, many reports have demonstrated continuous hyperglycemia to be the leading cause of diabetic nephropathy and that the normalization of hyperglycemia provides reno-protective effects. Therefore, we consider correction of hyperglycemia by canagliflozin to be involved in the protective effects observed in this study. In addition, we revealed that canagliflozin activates AMPK, and suppresses both cell proliferation and inflammation, independently of SGLT2 inhibition. We assume that unidentified mechanisms are also involved in the reno-protective effects exerted by canagliflozin. Further studies will be required to clarify this issue.

## Conclusions

It may be reasonable to consider normalized Pin1 expression and AMPK activation to be at least partially responsible for the molecular mechanisms underlying SGLT2 inhibitor-induced suppression of diabetic nephropathy.

## Data Availability

Not applicable.

## Abbreviations

2DG: 2-deoxy-d-glucose
ACC: acetyl-CoA carboxylase
ADP: adenosine diphosphate
AICAR: 5-aminoimidazole-4-carboxamide-1-β-d-ribofuranoside
AMPK: AMP-activated protein kinase
ATP: adenosine triphosphate
Cana: canagliflozin
C.C.: compound C
CTGF: connective tissue growth factor
DMEM: Dulbecco’s modified Eagle’s medium
eGFR: estimated glomerular filtration rate
HSD: high sucrose diet
KO: knock out
LPS: lipopolysaccharide
MCP-1: monocyte chemoattractant protein 1
mTOR: mammalian target of rapamycin
MTT: 3-(4,5-dimethylthiazol-2-yl)-2,5-diphenytletetrazolium bromide
ND: normal diet
NF-kB: nuclear factor-kappa B
PBS: phosphate buffered saline
PCR: polymerase chain reaction
PDGFRβ: platelet derived growth factor receptor-beta
Pin1: peptidylprolyl cis/trans isomerase, NIMA-interacting 1
PMSF: phenylmethylsulfonyl fluoride
PVDF: poly vinylidene di-fluoride
RPMI: Roswell Park Memorial Institute Medium
RT: room temperature
Ser: serine
SGLT2: sodium glucose co-transporter2
STZ: streptozotocin
TGF: transforming growth factor
Thr: threonine
TNFα: tumor necrosis factor-α
